# Variation in Corticosteroid Prescribing Practices for Patients With Septic Shock

**DOI:** 10.1097/CCE.0000000000001196

**Published:** 2025-02-21

**Authors:** Kanupriya Soni, John S. Minturn, Billie S. Davis, Leigh A. Bukowski, Jeremy M. Kahn, Ian J. Barbash

**Affiliations:** 1 Division of Pulmonary, Allergy and Critical Care Medicine, Department of Medicine, University of Pittsburgh, Pittsburgh, PA.; 2 Department of Critical Care Medicine, University of Pittsburgh School of Medicine, Pittsburgh, PA.; 3 Department of Health Policy and Management, University of Pittsburgh School of Public Health, Pittsburgh, PA.

**Keywords:** corticosteroids, critical care, intensive care, sepsis, septic shock

## Abstract

**OBJECTIVES:**

Understanding sources of variation in acute care delivery may inform targeted strategies to promote evidence-uptake. We sought to characterize physician-level and ICU-level variation in corticosteroid prescribing for patients with septic shock.

**DESIGN:**

We performed a retrospective cohort study using the electronic health record of a multihospital health system. We identified ICU patients with septic shock admitted between 2018 and 2020. Using medication administration data, we determined which patients received corticosteroids within 2 days of vasopressor initiation. We linked each patient to their attending physician of record using digital signatures from clinical documentation. We then fit a hierarchical mixed-effects logistic regression model to identify factors associated with corticosteroid use and quantify variation in corticosteroid administration across physicians and ICUs.

**SETTING:**

Twenty-six ICUs across nine hospitals in the United States.

**PATIENTS:**

ICU patients with septic shock.

**MEASUREMENTS AND MAIN RESULTS:**

Of 5322 patients with vasopressor dependent septic shock, 1294 (24.3%) were treated with corticosteroids within 2 days of vasopressor initiation. We linked these patients to 174 unique attending physicians across 26 ICUs. At the ICU-level, median corticosteroid use was 21.8% (interquartile range [IQR], 18.5–25.7%). At the physician-level, median corticosteroid use was 22.0% (IQR, 11.9–32.7%). In the mixed-effects regression controlling for patient and physician characteristics, 16.5% of the variation in corticosteroid administration was attributable to the ICUs and 10.1% was attributable to the physicians.

**CONCLUSIONS:**

Both ICUs and physicians contribute to observed variation in the use of corticosteroids for vasopressor dependent septic shock. These findings underscore the need for multilevel interventions to standardize evidence-based practices in critical care.

**KEY POINTS**

**Question:** What percentage of patients with vasopressor dependent septic shock receive corticosteroids, and to what degree do physicians and ICUs vary with respect to their corticosteroid prescribing practice patterns?

**Findings:** Only 24.3% of patients received corticosteroids within 2 days of vasopressor initiation, with substantial variation at the physician-level and ICU-level.

**Meaning:** Variation in corticosteroid prescribing practices for septic shock in the ICU stems from physicians and ICUs, indicating the need for multilevel interventions to standardize care of patients with septic shock.

Vasopressor dependent septic shock is present in about 10% of patients admitted to the ICU and is associated with in-hospital mortality rates approaching 40% (1). Although a hallmark of septic shock is uncontrolled systemic inflammation, few evidence-based treatments for septic shock target the inflammatory cascade that leads to organ dysfunction and death (2). The major exception to date is corticosteroids (3). Randomized trials suggest that corticosteroids can speed the resolution of shock, reduce organ dysfunction, and lower short-term mortality (4, 5). Yet, these beneficial effects are likely modest, and corticosteroids may also have deleterious side effects such as neuromuscular weakness, hyperglycemia, and superinfection (5).

In light of these tradeoffs, current clinical practice guidelines suggest using corticosteroids in patients with vasopressor dependent septic shock, while at the same time acknowledging that the risks may outweigh the potential benefits for some patients (6, 7). These guidelines leave considerable room for practice variation on the part of individual physicians. Practice variation in this setting may be desirable since in the absence of strong evidence physicians should be free to customize care based on their individualized assessments of the risks and benefits (8, 9). However, excessive variation, which might occur when similar patients are treated differently by different physicians, may represent a missed opportunity to standardize care, lower costs, and improve population-based outcomes in the ICU (10, 11).

To better understand this issue, we performed a retrospective cohort study of corticosteroid prescribing patterns for ICU patients with vasopressor dependent shock, focusing on physician-level and ICU-level variation in corticosteroid prescribing. To do so, we took advantage of a novel method for linking individual patients to specific ICU providers using metadata from the electronic health record (EHR) (12). Our study had two main objectives. First, we sought to determine the overall prevalence of corticosteroid use among patients with vasopressor dependent septic shock. Second, we sought to quantify variation in corticosteroid prescribing patterns across individual physicians and the ICUs in which they work. Our overall goal was to provide new insight into physician-level practice variation and help identify potential targets for standardizing practice and improving evidence-uptake in critical care.

## METHODS

### Study Design and Data

We performed a retrospective analysis of EHR data from UPMC, a multihospital academic and community health system in Western Pennsylvania. We included data from 26 ICUs across nine hospitals from 2018 to mid-2020. We selected these ICUs because they were staffed by intensivist physicians that round daily on each ICU patient, enabling us to establish a direct link between intensivists and individual patients (i.e., intensivist physicians rather than other individuals were responsible for decisions as to whether to prescribe corticosteroids). We selected this time period because it excludes the major surge of COVID-19 that occurred in the winter of 2020 in Western Pennsylvania.

Study ICUs were diverse in type, including not only medical, surgical, and mixed medical-surgical ICUs but also neurological ICUs. In addition, all included ICUs share a single EHR (Cerner PowerChart; Oracle Health, North Kansas City, MO) and contribute patient data to an ICU registry used for system-wide performance measurement and quality improvement. Data elements in the ICU registry include unique identifiers for each patient and hospital admission, patient demographics, ICU and hospital admission source, ICU and hospital discharge destination, vital signs, laboratory values, medication administration records, microbiology records, and respiratory care assessments including ventilator settings. Multiple prior studies used this registry to examine ICU patient outcomes, and complete details on the registry contents are available in those reports (13–16).

This research was reviewed and approved by the University of Pittsburgh Human Research Protections Office.

### Patient Population

We defined vasopressor dependent septic shock using the Sepsis-3 framework (13). Under this framework, we included patients if they were hospitalized in a study ICU with suspected infection and a requirement for vasopressor support. As in our past work, we defined suspected infection based on a blood culture draw and IV antibiotic administration within 24 hours before or after ICU admission, and we defined vasopressor support as receipt of a continuous infusion of norepinephrine, epinephrine, dopamine, or phenylephrine (15). We did not include patients who only received vasopressin without another vasopressor since use of vasopressin alone is uncommon and not necessarily indicative of a shock state (17).

### Physician Identification

We linked each patient-day to an individual attending physician using metadata from clinical documentation in the EHR (12). Whenever healthcare providers interact with a patient’s medical record, they create metadata in the form of electronic signatures indicating that they worked with the patient. For this study, we used metadata linked to clinical documentation (e.g., admission notes and progress notes). We identified all attending physicians that either wrote notes, cosigned notes, or addended notes on each individual patient-day, defined as the time interval between 7:00:00 am and 6:59:59 am the next day. We then used a deterministic algorithm to ascertain which of these individuals was the critical care attending physician of record, as previously described (12). The end result is a linkage file that assigns every patient a single attending physician of record on each day of their ICU stay. We supplemented these data with demographic characteristics on each attending physician obtained from the U.S. Centers for Medicare and Medicaid Services Doctors and Clinicians National Downloadable File.

### Variables

Our dependent variable was the administration of corticosteroids, specifically hydrocortisone, within 2 days of vasopressor administration. For simplicity, henceforth we refer to use of hydrocortisone within 2 days as “early corticosteroids.” We limited the corticosteroid of interest to hydrocortisone because it is the predominant steroid for this indication at our institution and the only corticosteroid studied in the randomized controlled trials that inform current guidelines (4–7); because the predominant use of hydrocortisone is for septic shock, whereas other corticosteroids such as dexamethasone, prednisone, and methylprednisolone are often used for other indications such as obstructive lung disease (18); and because fludrocortisone is most often given as an adjunct to hydrocortisone rather than a stand-alone drug for this indication. We assessed early corticosteroid use both at the level of the patient (i.e., whether or not that patient received early corticosteroids) and at the level of the physician (i.e., the percentage of patients treated with early corticosteroids out of the total number of patients with vasopressor dependent septic shock treated by each physician).

We also calculated the maximum dose of vasopressors within 2 days of vasopressor initiation for patients who received early corticosteroids. As with early corticosteroid use, we defined this variable for patients (i.e., the maximum vasopressor dose within 2 days of vasopressor initiation) and physicians (i.e., the median vasopressor dose across patients treated with early corticosteroids per physician). To facilitate comparisons, we standardized vasopressor dosing into norepinephrine equivalents (19).

Additional variables included patient-level and physician-level factors hypothesized to influence corticosteroid prescribing. Patient-level variables included age, sex, ICU admission source (categorized as emergency department, operating room, procedure unit, intermediate care, ward, other, or missing), surgical status (defined as a major surgery before or at onset of vasopressor administration), mechanical ventilation (defined as receipt of invasive mechanical ventilation at onset of vasopressor administration), and Sequential Organ Failure Assessment (SOFA) score at the time of vasopressor initiation. Physician-level variables included sex, experience (defined as years after medical school graduation), base specialty (categorized as internal medicine without pulmonary training, internal medicine with pulmonary training, or other, with the other group including anesthesia, surgery, emergency medicine, and neurology), and caseload (number of unique vasopressor dependent septic shock cases seen during the study period). We also make note of ICU characteristics (ICU type, number of beds per ICU, physician staffing model, and number of patients with vasopressor dependent septic shock over the study period) and distribution of patients treated with early corticosteroids.

### Analysis

We summarized patient and physician characteristics using standard descriptive statistics. For patients, we categorized them into two groups based on whether or not they received early corticosteroids. We compared the groups using Wilcoxon rank-sum tests and chi-square tests, as appropriate. For physicians, we categorized them into four quartiles based on the percentage of patients that they treated with early corticosteroids. We compared the quartiles using Kruskal-Wallis tests and chi-square tests, as appropriate. To avoid low-volume outliers overly influencing the results, in making these comparisons, we limited the analysis to only physicians seeing ten or more patients over the study period.

To visualize physician-level variation in prescribing patterns, we created a caterpillar plot showing the percentage of patients that each physician treated with early corticosteroids also limiting to physicians seeing ten or more patients over the study period. We estimated 95% CIs for each percentage using the binomial distribution. We also assessed the relationship between vasopressor dose and frequency of early corticosteroid administration by physicians. To graphically visualize this distribution, we plotted physicians in rank order by percentage of patients each physician treated with early corticosteroids along the *X*-axis. Along the *Y*-axis, we plotted the median vasopressor dose among all patients treated with corticosteroids by each physician. The goal of this analysis was to understand if physicians that tended to prescribe early corticosteroids with greater frequency tended to prescribe corticosteroids at lower vasopressor doses. We tested this relationship using the Spearman rank correlation coefficient.

To quantify physician-level and ICU-level variation in corticosteroid prescribing, we fit a hierarchical mixed-effects logistic regression model in which the dependent variable was whether or not the patient received early corticosteroids. The model included patient-level and physician-level factors as well as random effects for the physician and the ICU. We used this model to: 1) identify the patient-level factors associated with corticosteroid prescribing, controlling for any potential influence of the physician and ICU and 2) estimate the physician-level and ICU-level random effects.

The model is based on a non-nested structure. In cases in which a single attending physician worked in multiple ICUs, we treated those individuals as different physicians. This approach is conservative since it would serve to reduce observed variation across providers.

In addition, we performed a sensitivity analysis looking at corticosteroid prescription within a longer time frame of 4 days from vasopressor initiation. We limited the sensitivity analysis to 4 days because initiation of corticosteroids beyond that time frame likely indicates treatment in response to a clinical change (i.e., new infection).

To quantify physician-level and ICU-level random effects we calculated intraclass correlation coefficients and median odds ratios (ORs). Intraclass correlation coefficients indicate how much of the variance in prescribing practices are attributable to the cluster (i.e., physician or ICU), relative to the total variance in the model (20). Median ORs indicate the median value of the OR comparing a higher-use cluster to a lower-use cluster when two clusters are selected at random (21). This value can be interpreted as the increase in the odds of early corticosteroid receipt had the patient been treated by a different higher-use cluster, holding all other model factors constant. The median OR is similar conceptually to the intraclass correlation coefficient, but may be more intuitive because it measures the influence of cluster on a log-odds scale, enabling direct comparisons to other variables in the model. We also graphically depict the physician-specific and ICU-specific effect sizes to assess differences in degree of variation.

Data management was performed using Microsoft SQL Server Management Studio, Version 18.11.1 (Microsoft Corporation, Redmond, WA) and Stata, Version 18.0 (StataCorp, College Station, TX). The statistical analysis was performed using Stata, Version 18.0 (StataCorp) and R, Version 4.2.0 (R Foundation, Ames, IA). The mixed-effects model was fit using the lem4 package in R (R Foundation). A *p* value of below 0.05 was considered significant.

## RESULTS

We identified 5322 patients with vasopressor dependent septic shock, of whom 1294 (24.3%) were treated with a corticosteroid within 2 days of vasopressor administration (**Table 1**). Patients who did and did not receive early corticosteroids were similar in age, sex, ICU admission source, surgical status, and mechanical ventilation. Patients who received early corticosteroids were sicker, as evidenced by a higher mean SOFA score (7.3 ± 4.1 vs. 6.1 ± 3.3; *p* < 0.01), a higher median maximum vasopressor dose (0.50 vs. 0.20 µg/kg/min; *p* < 0.01), and higher in-hospital mortality (45.7% vs. 32.5%; *p* < 0.01). Patients who received early corticosteroids had shorter hospital lengths of stay (14.0 ± 15.3 vs. 14.9 ± 15.2; *p* < 0.01) and shorter ICU lengths of stay (6.9 ± 7.2 vs. 7.5 ± 8.6; *p* = 0.02).

**TABLE 1.** Patient Characteristics by Group

Characteristic	Early Corticosteroids (*n* = 1294)	No Early Corticosteroids (*n* = 4028)	*p* ^a^
Age, mean ± sd	64.8 ± 14.5	65.4 ± 15.0	0.17
Sex			
Female	634 (49.0%)	1902 (47.2%)	0.28
Male	660 (51.0%)	2126 (52.8%)	
ICU admission source			
Emergency department	712 (55.0%)	2196 (54.5%)	0.39
Operating room	18 (1.4%)	75 (1.9%)	
Procedure unit	39 (3.0%)	99 (2.5%)	
Intermediate care	148 (11.4%)	419 (10.4%)	
Ward	178 (13.8%)	533 (13.2%)	
Other	155 (12.0%)	546 (13.6%)	
Missing	44 (3.4%)	160 (4.0%)	
Surgical status = yes	235 (18.2%)	804 (20.0%)	0.17
Mechanical ventilation = yes	769 (59.4%)	2280 (56.6%)	0.08
Length of mechanical ventilation, d, mean ± sd	4.2 ± 6.6	4.5 ± 8.0	0.12
Sequential Organ Failure Assessment score, mean ± sd	7.3 ± 4.1	6.1 ± 3.3	< 0.01
Maximum vasopressor dose in µg/kg/min of norepinephrine equivalents, median (interquartile range)^b^	0.5 (0.2–1.1)	0.2 (0.1–0.5)	< 0.01
ICU length of stay, d, mean ± sd	6.9 ± 7.2	7.5 ± 8.6	0.02
Hospitalization length of stay, d, mean ± sd	14.0 ± 15.3	14.9 ± 15.2	< 0.01
In-hospital mortality	591 (45.7%)	1308 (32.5%)	< 0.01

a The *p* values are from Wilcoxon rank-sum tests for continuous variables and χ^2^ test for categorical variables.

b Maximum reported dose within 2 d of vasopressor initiation.

Values are frequency (percent) unless otherwise noted.

We identified 174 physicians who treated patients with vasopressor dependent septic shock.

**Figure 1.**
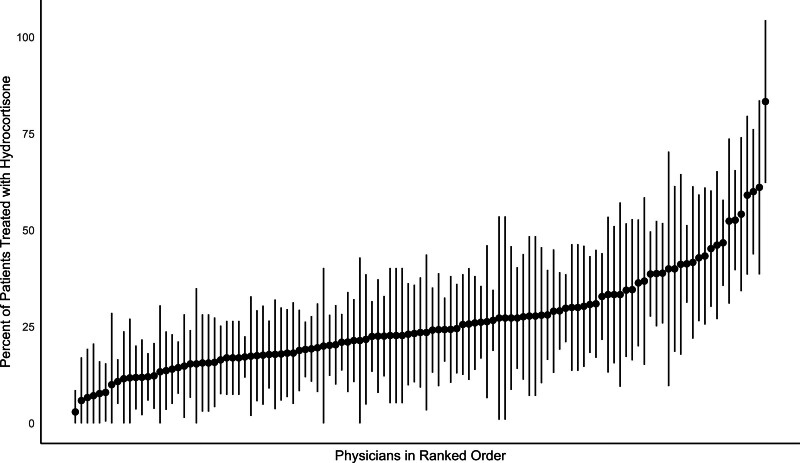
Physician-level variation in administration of early corticosteroids in septic shock. Each *black dot* denotes an individual attending physician (*n* = 115) ranked in increasing order by percentage of patients treated with early corticosteroids. The *Y*-axis shows the percentage of patients treated with early corticosteroids and the associated 95% CIs. This includes only physicians seeing ten or more patients over the study period.

**Figure 2.**
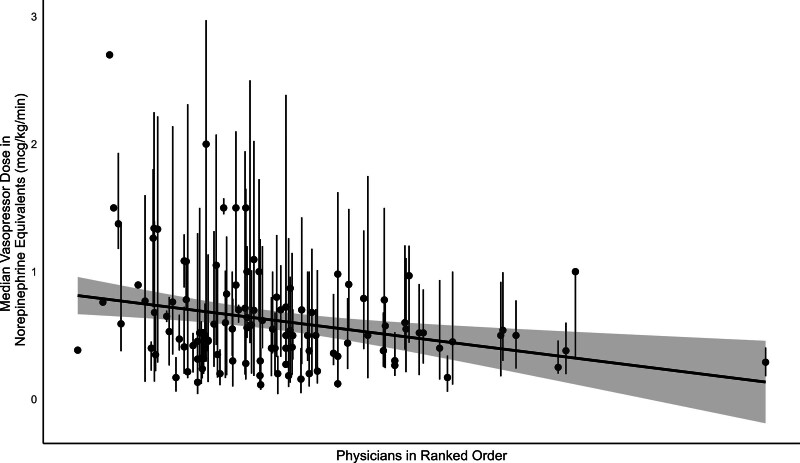
Relationship between physician administration of early corticosteroids in septic shock and vasopressor dose. Each *black dot* denotes an individual attending physician (*n* = 115) ranked on the *X*-axis in increasing order by percentage of patients treated with early corticosteroids. The *Y*-axis shows the median vasopressor dose in norepinephrine equivalents (µg/kg/min) among all patients treated with corticosteroids by each physician. An individual patient’s vasopressor dose represents the maximum vasopressor dose within 2 d of vasopressor initiation. The Spearman rank correlation coefficient is –0.24 (*p* < 0.01). This includes only physicians seeing ten or more patients over the study period.

There was wide variation in early corticosteroid prescription by physician, from none to all patients. The median percentage was 22.0% (interquartile range, 11.9–32.7%). When limiting the cohort to only physicians treating more than ten patients (*n* = 115), there were no significant differences in characteristics between high prescribers and low prescribers (**Table 2**). Of the physicians who treated more than ten patients (*n* = 115), the percentage of patients treated with early corticosteroids ranged from 2.9% to 83.3% (**Fig. 1**). Physicians that prescribed corticosteroids more frequently also did so at a lower dose of vasopressors (**Fig. 2**).

**TABLE 2.** Physician^a^ Characteristics by Rate of Early Corticosteroid Prescribing, Categorized by Quartile

Characteristic	Quartile 1 (*n* = 29)	Quartile 2 (*n* = 28)	Quartile 3 (*n* = 29)	Quartile 4 (*n* = 29)	*p* ^b^
Percent treated with early corticosteroids					
Range	2.9–17.2%	17.4–23.3%	23.5–30.8%	31.0–83.3%	Not applicable
Median (IQR)	13.3% (10.8–15.6%)	20.3% (18.2–22.5%)	27.3% (25.6–28.1%)	41.2% (36.4–46.8%)	
Sex					
Female	10 (34.5%)	6 (21.4%)	5 (17.2%)	9 (31.0%)	0.40
Male	19 (65.5%)	22 (78.6%)	24 (82.8%)	20 (69.0%)	
Experience (yr)					
< 10	1 (3.5%)	0 (0.0%)	0 (0.0%)	0 (0.0%)	0.46
10–19	15 (51.7%)	12 (42.9%)	10 (34.5%)	9 (31.0%)	
20–29	9 (31.0%)	11 (39.3%)	9 (31.0%)	12 (41.4%)	
30 +	4 (13.8%)	5 (17.9%)	10 (34.5%)	8 (27.6%)	
Base specialty					
IM, not pulmonary	13 (44.8%)	11 (39.3%)	10 (34.5%)	14 (48.3%)	0.15
IM, pulmonary	11 (37.9%)	13 (46.4%)	18 (62.1%)	8 (27.6%)	
Other^c^	5 (17.2%)	4 (14.3%)	1 (3.5%)	7 (24.1%)	
Caseload^d^					
Range	10–192	14–119	11–120	10–92	0.21
Median (IQR)	42.0 (26.0–59.0)	39.5 (26.8–59.0)	34.0 (25.0–53.0)	27.0 (21.0–42.0)	

IM = internal medicine, IQR = interquartile range.

a This includes only physicians seeing ten or more patients over the study period.

b The *p* values are from Kruskal-Wallis tests for continuous variables and χ^2^ tests for categorical variables.

c “Other” category for base specialty includes anesthesia, emergency medicine, surgery, and neurology.

d Number of unique vasopressor dependent septic shock patients seen during the study period.

Values are frequency (percent) unless otherwise noted.

Across the 26 study ICUs, the percentage of patients treated with early corticosteroids ranged from 9.5% to 46.2% with median and interquartile range of 21.8% (18.5–25.7%). Additional ICU-level characteristics can be found in **Table E1** (http://links.lww.com/CCX/B452).

The results of the hierarchical mixed-effects logistic regression model are shown in **Table 3**. Surgical status was associated with a significantly decreased odds of receiving early corticosteroids (OR, 0.81; 95% CI, 0.66–0.98; *p* = 0.03), while SOFA score was associated with an increased odds of receiving early corticosteroids (OR, 1.10; 95% CI, 1.07–1.12; *p* < 0.01). The median OR for physicians (OR, 1.35; 95% CI, 1.31–1.40) and ICUs (OR, 1.48; 95% CI, 1.36–1.61) were larger than any fixed effect. A sensitivity analysis investigating corticosteroid administration with an expanded time frame to 4 days after initiation of vasopressors showed similar results (**Table E2**, http://links.lww.com/CCX/B452). Only an additional 182 (4.5%) of the 4028 patients who did not receive early corticosteroids were ultimately treated with corticosteroids within 4 days of vasopressor initiation.

**TABLE 3.** Factors Associated With Treatment With Early Corticosteroids in Septic Shock^a^

Factors	OR (95% CI)	*p*
Patient-level factors		
Age	1.00 (1.00–1.01)	0.33
Female	1.03 (0.89–1.18)	0.70
ICU admission source		
Emergency department	Referent	NA
Operating room	1.22 (0.69–2.15)	0.49
Procedure unit	1.05 (0.68–1.62)	0.83
Intermediate care	0.94 (0.75–1.18)	0.59
Ward	0.99 (0.81–1.22)	0.95
Other	0.93 (0.74–1.17)	0.55
Missing	0.72 (0.49–1.04)	0.08
Surgical status = yes	0.81 (0.66–0.98)	0.03
Mechanical ventilation = yes	0.87 (0.74–1.02)	0.08
Sequential Organ Failure Assessment score	1.10 (1.07–1.12)	< 0.01
Physician-level factors		
Female	1.07 (0.84–1.35)	0.58
Experience (yr)	1.00 (0.99–1.01)	0.50
Base specialty		
IM, not pulmonary	Referent	NA
IM, pulmonary	1.12 (0.88–1.44)	0.35
Other	1.05 (0.76–1.45)	0.77
Caseload^b^	0.99 (0.96–1.02)	0.41
Physician-level median OR	1.35 (1.31–1.40)	NA
ICU-level median OR	1.48 (1.36–1.61)	NA

IM = internal medicine, NA = not applicable, OR = odds ratio.

a The table shows the results of the hierarchical mixed-effects logistic regression model that includes the listed patient and physician factors, as well as random effects at the level of the physician and the ICU.

b For each ten patient-increase in vasopressor dependent septic shock patients seen during the study period.

**Figure 3.**
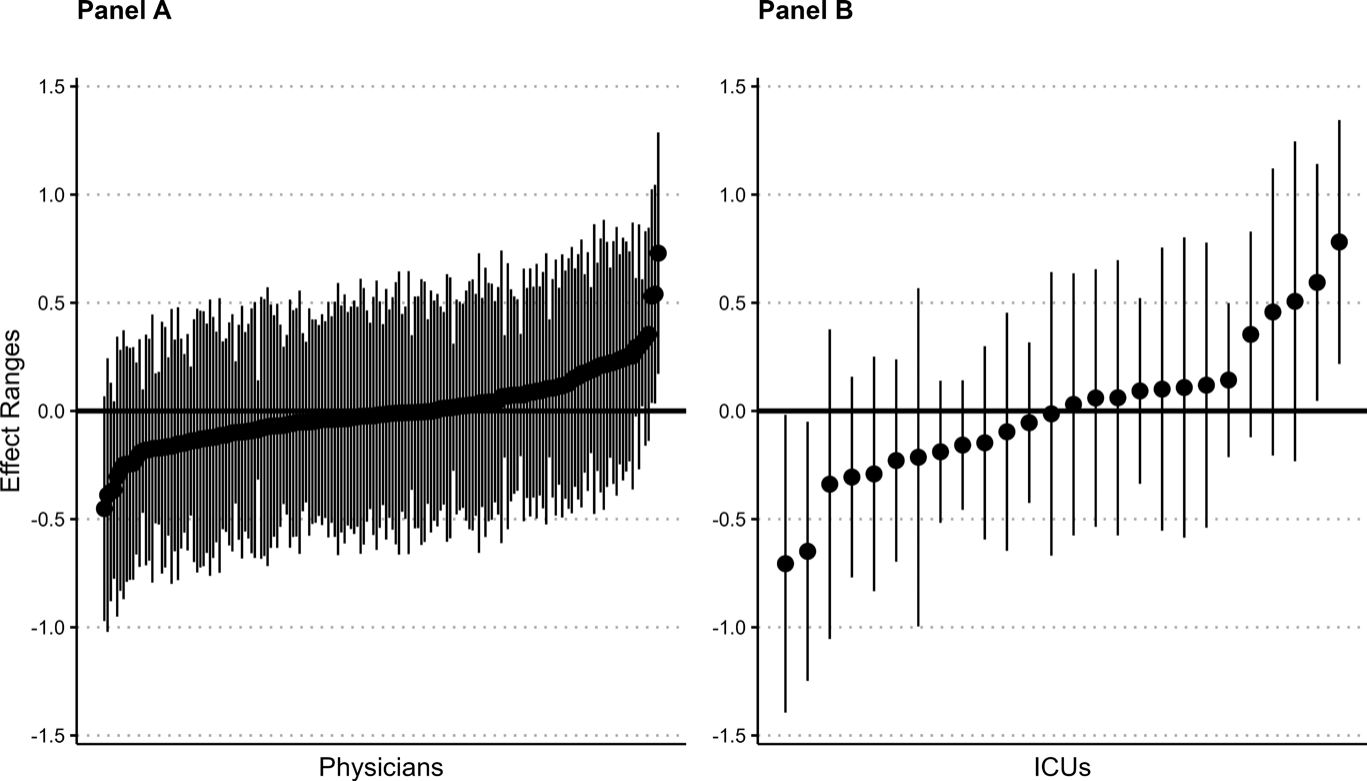
Estimates of physician-level and ICU-level random effects from the hierarchical mixed-effects logistic regression model. Each *black dot* represents a physician (**A**, *n* = 174) or ICU (**B**, *n* = 26) and the degree of variation in administration of early corticosteroids compared with the median effect across all physicians or ICUs, respectively.

In the main model, physicians constituted 10.1% of the total model variance and ICUs accounted for 16.5% of the total model variance. The intraclass correlation for physicians was 0.03 (95% CI, 0.01–0.05) and the intraclass correlation for ICUs was 0.05 (95% CI, 0.01–0.08). The graphical representation of physician-specific and ICU-specific effect sizes from the model depict a wider observed variation by ICU compared with by physician (**Fig. 3**).

## DISCUSSION

Employing a novel method for linking individual intensivist physicians to ICU patients using EHR metadata, we quantified variation in early corticosteroid prescribing in patients with vasopressor dependent septic shock. We demonstrated significant variation in this practice at both the physician-level and the ICU-level, even after controlling for patient and physician characteristics. Although there was substantial variation at both levels, the identity of the ICU accounted for slightly more variation than the identity of the physician. These findings suggest the presence not only of physician-specific practice styles when it comes to prescribing corticosteroids in septic shock but also ICU-specific behavioral norms that influence prescribing over and above individual clinicians.

These findings have several implications for efforts to reduce variation and standardize practice in critical care. Specifically, our results underscore the importance of deploying multilevel interventions in the effort to improve evidence-uptake, particularly in scenarios when the evidence is not definitive. Neither physician-level interventions, such as online education, nor ICU-level interventions, such as clinical protocols, are likely to be influential by themselves (22). Rather, interventions to promote practice standardization are more likely to succeed when they target all the sources of variation. More broadly, our results highlight the inherent difficulty in standardizing practice in the setting of nondefinitive evidence, given that we observed over ten-fold variation in steroid use across physicians.

Our study also provides new insight into the circumstances by which physicians opt to treat patients in septic shock with corticosteroids. Even accounting for physician-level and ICU-level variation, patients were more likely to receive early corticosteroids when they had greater severity of illness. In addition, physicians that tended to prescribe corticosteroids less tended to prescribe them for patients with higher vasopressor doses. These findings suggest that physicians are employing a representativeness heuristic in corticosteroid decision-making. Representative heuristics occur when decision makers form judgments about the likelihood of an event based on how similar the case is to a preconceived prototype (23). In this case, it appears that physicians perceive a higher likelihood that the patient will benefit from corticosteroids when the patients is more evidently in a severe shock state.

In contrast, other studies point to the lack of a significant availability heuristic in clinical decision-making. For example, in a study of lung-protective ventilation use in patients with acute respiratory distress syndrome, Bellani et al (24) reported no differences in tidal volumes based on the severity of lung injury. It is possible that this difference is explained by differences in the strength of evidence underlying these two practices or differences in the way these two practices are delivered, in that lung-protective ventilation is a complex practice that requires a multidisciplinary care team to implement, and therefore is less dependent on individual decision-making, whereas corticosteroids require only a medication order and therefore may be more susceptible to physician-level biases.

Last, our results reveal an ongoing implementation gap, in that only 24.3% of patients with vasopressor dependent septic shock received early corticosteroids. This result is similar to other multicenter studies in the United States, which report corticosteroid prescribing rates from 23% to 27.6% (25, 26). However, it is lower than reported in some single center studies and some international studies, in which corticosteroid prescribing rates range from 35.0% to 53.0% (27–29). It is unlikely that all patients with vasopressor dependent septic shock can or should receive corticosteroids, as underscored by clinical practice guidelines in which the steroid recommendation is graded as “conditional” due to the lack of definitive evidence and potential tradeoffs involved. Nonetheless, under the Grading of Recommendations, Assessment, Development, and Evaluations framework, even a conditional recommendation implies that the majority of individuals with septic shock should receive corticosteroids (30). Instead, we observed only a small minority receiving corticosteroids, indicating an important gap between current guidelines and clinical practice.

Our study has several limitations. First, our data are from a single health system in one region, so our findings may not generalize to other settings. However, the health system includes a large number of hospitals with varying characteristics including size, urban setting, teaching hospital, and patient case-mix index. Second, this study focuses on attending physicians. In practice, ICU teams include clinicians of different backgrounds such as residents, fellows, advanced practice providers, and clinical pharmacists, all of whom may influence decisions to prescribe corticosteroids in septic shock. For practical reasons, we assigned ultimate responsibility for medical decisions and orders to attending physicians, but we acknowledge that more sophisticated approaches accounting for complex decision-making among team members may yield different insights. More broadly, it is conceivable that corticosteroids prescribed during off hours or by providers who are not the attending of record do not reflect the attending physician’s actual practice pattern, although we expect this occurrence to be rare. Third, we could not completely exclude the possibility that patients received hydrocortisone for indications other than septic shock or that physicians prescribed steroids other than hydrocortisone for septic shock, although we expect these instances to be rare.

## CONCLUSIONS

We used EHR metadata to capture variation in corticosteroid prescribing in septic shock at the level of individual intensivists and ICUs. This approach may inform future work to more effectively tailor knowledge translation interventions for acutely ill patients.

## Supplementary Material


