# Supplementary material for: Variation in Corticosteroid Prescribing Practices for Patients With Septic Shock
Source: Crit Care Explor. 2025 Feb 21;7(3):e1196. doi: 10.1097/CCE.0000000000001196 (PMC11845208; doi:10.1097/CCE.0000000000001196)
Supplement: Supplementary file 1 [file cc9-7-e1196-s001.pdf]

Supplementary Material

Variation in Corticosteroid Prescribing Practices for Patients with Septic Shock

Table E1. Intensive care unit (ICU) characteristics (n = 26)

Characteristic	Value
ICU type	
Cardiac/cardiothoracic surgery	4 (15.4%)
Medical	4 (15.4%)
Mixed medical/surgical	8 (30.8%)
Neurological	3 (11.5%)
Surgical	7 (26.9%)
Number of Beds	
Median<br>[IQR]	14<br>[8 - 20]
Physician staffing model	
Closed	18 (69.2%)
Mandatory intensivist consult	8 (30.8%)
Open	0 (0.0%)
Patients with vasopressor dependent septic shock	
Median<br>[IQR]	155<br>[112 - 289]
Patients treated with early corticosteroids	
Range	9.5% - 46.2%
Median<br>[IQR]	21.8%<br>[18.5% - 25.7%]

Values are frequency (percent) unless otherwise noted.

IQR = interquartile range

Table E2. Factors associated with treatment with corticosteroids in septic shock, within four days of vasopressor initiation[a]

	**Odds Ratio (95% CI)**	**p-value**
**Patient-level factors**		
Age	1.00 (1.00 - 1.01)	0.28
Female	1.02 (0.89 - 1.16)	0.80
ICU admission source		
Emergency department	Referent	-
Operating room	1.33 (0.78 - 2.26)	0.29
Procedure unit	1.06 (0.70 - 1.61)	0.78
Intermediate care	0.90 (0.72 - 1.12)	0.34
Ward	0.99 (0.82 - 1.21)	0.94
Other	0.94 (0.75 - 1.17)	0.57
Missing	0.76 (0.54 - 1.09)	0.13
Surgical status = yes	0.83 (0.69 - 1.00)	0.06
Mechanical ventilation = yes	0.88 (0.75 - 1.02)	0.09
SOFA score	1.10 (1.08 - 1.13)	<0.01
**Physician-level factors**		
Female	1.04 (0.83 - 1.29)	0.76
Experience (years)	1.01 (1.00 - 1.02)	0.24
Base specialty		
IM, not pulmonary	Referent	-
IM, pulmonary	1.11 (0.88 – 1.40)	0.39
Other	1.02 (0.75 – 1.39)	0.89
Caseload[b]	0.99 (0.96 – 1.02)	0.42
**Physician-level median OR**	1.31 (1.28 – 1.36)	--
**ICU-level median OR**	1.52 (1.39 – 1.64)	--

CI = confidence interval; ICU = intensive care unit; IM = internal medicine; SOFA = sequential organ failure assessment

[a]The table shows the results of the hierarchical mixed effects logistic regression model that includes the listed patient and physician factors, as well as random effects at the level of the physician and the ICU. Of 5,322 patients, 1,476 (27.7%) received corticosteroids within four days of vasopressor initiation.

[b]For each 10 patient-increase in vasopressor dependent septic shock patients seen during the study period.
